# Portable and Rapid Smartphone-Based Colorimetric Assay of Peracetic Acid for Point-of-Use Medical/Pharmaceutical Disinfectant Preparation

**DOI:** 10.3390/molecules30132798

**Published:** 2025-06-28

**Authors:** Suphakorn Katib, Sutasinee Apichai, Jutamas Jiaranaikulwanitch, Busaban Sirithunyalug, Fumihiko Ogata, Naohito Kawasaki, Kate Grudpan, Chalermpong Saenjum

**Affiliations:** 1 Department of Pharmaceutical Sciences, Faculty of Pharmacy, Chiang Mai University, Chiang Mai 50200, Thailand; suphakorn2801@gmail.com (S.K.); jutamas.jia@cmu.ac.th (J.J.); busaban.s@cmu.ac.th (B.S.); 2 Research Center for Innovation in Analytical Science and Technology for Biodiversity Based Economic and Society (I-ANALY-S-T_B.BES-CMU), Chiang Mai University, Chiang Mai 50200, Thailand; sutasinee.apichai@cmu.ac.th (S.A.); kgrudpan@gmail.com (K.G.); 3 Office of Research Administration, Chiang Mai University, Chiang Mai 50200, Thailand; 4 Faculty of Pharmacy, Kindai University, 3-4-1 Kowakae, Higashi-Osaka 577-8502, Osaka, Japan; ogata@phar.kindai.ac.jp (F.O.); kawasaki@phar.kindai.ac.jp (N.K.); 5 Antiaging Center, Kindai University, 3-4-1 Kowakae, Higashi-Osaka 577-8502, Osaka, Japan; 6 Department of Chemistry, Faculty of Science, Chiang Mai University, Chiang Mai 50200, Thailand

**Keywords:** peracetic acid, disinfectant, on-site analysis, colorimetric method, digital image-based method, smartphone

## Abstract

A simple and rapid smartphone-based colorimetric assay for peracetic acid concentration was developed to facilitate point-of-use disinfectant preparations for infection prevention and control. The colorimetric detection was based on the oxidation of N,N-diethyl-phenylenediamine by peracetic acid through an intermediate reaction with potassium iodide, resulting in pink-magenta products. The colorimetric reaction was performed on a 96-well plate; then, the color products were photographed in one image. The color intensity was evaluated to determine the peracetic acid concentration using a custom-built mobile application named Modern Peracetic Acid Analysis. The relative green intensity of the pink-magenta products was directly proportional to the peracetic acid concentration in the range of 0.15 to 3.0 µg/mL. The detection and quantitation limits were 0.11 µg/mL and 0.34 µg/mL, respectively. The approach was successfully applied to determine the peracetic acid concentration in pharmaceutical disinfectant formulations. The results obtained using the proposed approach showed no significant differences from those obtained using acid–base titration at the 95% confidence level. The greenness of the proposed approach was evaluated using the Complementary Green Analytical Procedure Index, Analytical Greenness, and Blue Applicability Grade Index, demonstrating enhanced environmental friendliness and practical advantages, as well as simple, portable instrumentation that is easier to operate than traditional spectrophotometric and titration methods. Furthermore, a sustainability assessment based on the Need, Quality, and Sustainability index underscored its enhanced sustainability.

## 1. Introduction

Peracetic acid (PAA) is a potent, sterile, antimicrobial, oxidizing agent extensively used for infection prevention and control (IPC). Its high efficacy in oxidizing proteins and lipids, which leads to the denaturation of cell walls and cellular DNA, enhances its appeal as a promising alternative oxidant and disinfectant [1]. It is widely used in hospitals, healthcare, and pharmaceutical facilities; the food and beverage industries; and in livestock contexts. Typically, PAA is provided to users in concentrated solutions (25–40%). Therefore, preparation processes are needed to achieve ready-to-use concentrations. Regarding human hygiene, the Centers for Disease Control and Prevention recommends various concentration levels; however, concentrations of 0.2% or higher are sporicidal. The PAA immersion system functions at 50–56 °C to decontaminate exterior surfaces, lumens, and medical accessories [2]. In addition, PAA is registered as a biocidal active substance under the Biocidal Products Regulation (EU) 528/2012 [3]. It is approved for use in veterinary hygiene biocidal products, food and feed area disinfectants, drinking water disinfectants, and in-can preservatives. Various peracetic acid concentrations are used depending on the application. For example, concentrations ranging from 20 to 3000 ppm are employed in automated spraying systems for aseptic filling and the sterilization of livestock, cheese molds, and food crates in the food and beverage industry. Concentrations between 50 and 250 ppm are used to disinfect milking parlors, while 25 ppm is applied to disinfect animal drinking water. In addition to its standalone application, PAA is also used in combination with other agents in various mixtures. For example, in a previous study, a mixture of PAA and sodium hypochlorite was used to disinfect surfaces contaminated with SARS-CoV-2 [4]. Similarly, a combination of PAA with acetic acid (AcOH) was applied for the rinsing-free disinfection of cherry tomatoes [5], and a mixture of PAA and 2-hydroxyphenyl was employed to reduce the spread of antimicrobial resistance in weaned pig barns [6]. PAA has been utilized across a wide range of concentrations and applications, necessitating the preparation of various ready-to-use formulations. To ensure effective sterilization and to maintain safety standards, the ready-to-use concentration must be verified before each application.

There are various techniques for PAA determination, such as titration [7], chromatographic [8], electrochemical [9], fluorometric [10], and spectrophotometric methods [11,12]. However, these laboratory techniques are unsuitable for applications that require rapid, facilitated on-site analyses and low costs per sample. Although portable devices [13,14] and commercial test kits [15] are available for rapid on-site analyses, they are often costly, or the results are estimated visually. An alternative method based on colorimetric image processing that employs a specific colorimetric reaction has gained attention for its potential development as a rapid, on-site analysis technique [16]. Several colorimetric reactions have been proposed for PAA determination, such as the Tringer reaction, which produces a red anthraquinone compound [17,18], and the oxidation of bis(3-ethylbenzothiazoline-6-sulfonate) (ABTS), which forms green free radical cations (ABTS^•+^) [11]. The N,N-diethyl-p-phenylenediamine (DPD) reaction is preferred and accepted by the US Environmental Protection Agency for the determination of PAA concentrations [19]. The main oxidation product of the DPD-PAA reaction at near-neutral pH is a semiquinone-type cationic compound known as Würster dye, which exhibits a pink-magenta color [12,20]. Additionally, the reaction proceeds rapidly and exhibits selectivity for PAA, even in the presence of other oxidants, making it highly suitable for practical applications. By integrating image processing technology with smartphone-based colorimetric detection, this reaction enables the development of a portable and user-friendly assay for point-of-use scenarios, particularly in medical and pharmaceutical disinfectant preparation.

The aim of this work was to develop a simple, rapid, and green alternative method for PAA determination with the colorimetric DPD reaction. A portable analytical system that integrates a multi-well plate platform with a smartphone detector and a custom mobile application was developed to enable convenient on-site analyses. The determination of PAA in pharmaceutical disinfectants was demonstrated as an initial model for real-world application, promoting sustainable pharmaceutical analyses, especially for IPC. Furthermore, this approach can be extended to the food and beverage industries, as well as cattle farming, to support sustainable practices in both agroindustry and livestock.

## 2. Results and Discussion

### 2.1. Spectrophotometric and Image Processing Investigation of the DPD-PAA Reaction

**Figure 1:**
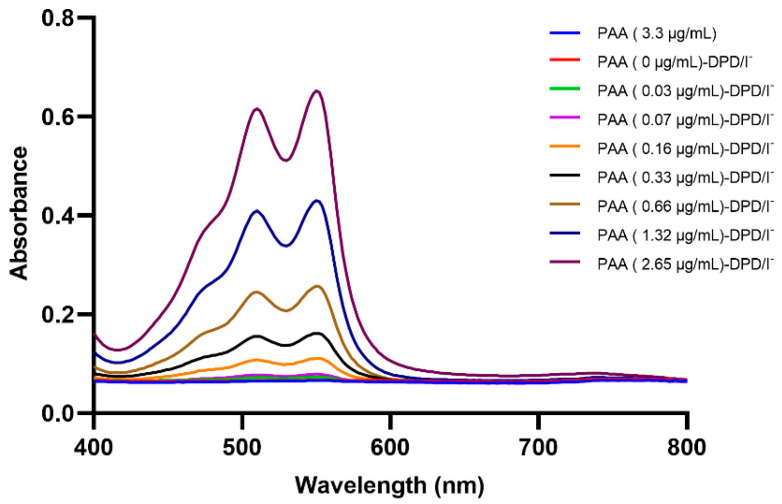
Absorption spectra of DPD oxidation with different PAA concentrations. Experimental conditions: [PAA] = 0–2.65 µg/mL, [DPD] = 1.1 mM, [AcOH] = 4.3 mM, [I^−^]_initial_ = 26.1 mM, and reaction time = 60 s.

The colorimetric reaction proposed for PAA determination was adapted from the Chhetri method [21]. The absorption spectrum of the pink-magenta products was measured between 400 nm and 800 nm. Absorption maxima were detected at 515 nm and 550 nm (Figure 1), which were absorption of Würster dyes (DPD^•+^). This is consistent with other reports, including Chhetri et al. [21], Liu et al. [22], and Li et al. [23], who reported that the absorption peaks of Würster dyes are at 515 nm and 551 nm. Cheng et al. also reported that 512 nm and 553 nm could be used for PAA determination [18]. The results showed that the product absorbance increased in correlation with the PAA concentration because PAA (CH_3_CO_3_H) rapidly reacts with potassium iodide (KI) to produce iodine (I_2_) [24,25], and I_2_ then selectively oxidizes DPD to DPD^•+^ (the pink-magenta product), which is called Würster dye, as shown in Equations (1) and (2) [12,18,26]:

CH_3_CO_3_H + 2I^−^ + 2H^+^ → I_2_ + CH_3_COOH + H_2_O (1)

(2) $$\mathrm{DPD}\;\overset{I_{2}}{\to }\;{\mathrm{DPD}}^{•+}\;(\mathrm{pink}-\mathrm{magenta})$$

The correlation between the color intensity of the pink-magenta product and the PAA concentration was investigated using the image processing technique. The red (R), green (G), and blue (B) intensities of the pink-magenta product obtained using PAA concentrations from 0 to 2.65 µg/mL were determined. The relative intensity (Δ intensity) of each color was plotted against the PAA concentration and was examined using Pearson correlation. Among the colors in R-G-B mode, the green intensity exhibited a wide linear range and the highest sensitivity with a high Pearson correlation coefficient (see Figure S1). This indicated that the Δ green intensity could be used for PAA determination.

### 2.2. Investigation of the Optimum Conditions for PAA Determination

#### 2.2.1. Effect of AcOH Concentration on Product Formation in PAA Determination

**Figure 2:**
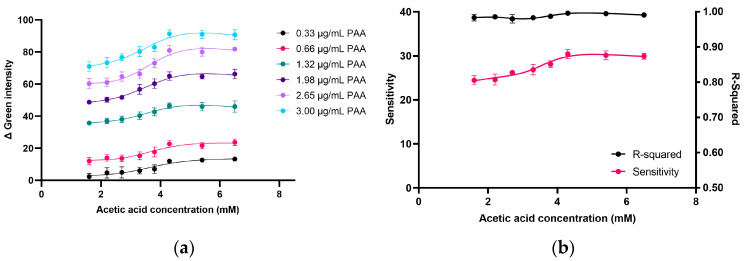
Effects of acetic acid concentration on (**a**) ∆ green intensity and (**b**) sensitivity and linear correlation (R-squared) of calibration graph. Experimental conditions: [PAA] = 3.31 µg/mL, [DPD] = 1.1 mM, [AcOH] = 1.6, 2.2, 2.7, 3.3, 3.8, 4.3, 5.4, and 6.5 mM, [I^−^]_initial_ = 26.1 mM, and reaction time = 60 s (*n* = 3).

In addition to AcOH’s role in maintaining the balance between PAA and hydrogen peroxide (H_2_O_2_), it also affects the solution’s pH. This pH adjustment is critical, as it affects iodide (I^−^) oxidation, a key step in PAA determination, as shown in Equation (1) [21]. Thus, the effect of the AcOH concentration (1.6, 2.2, 2.7, 3.3, 3.8, 4.3, 5.4, and 6.5 mM) on the analytical characteristics, including the sensitivity (the slope of the calibration graph) and linear correlation, were investigated, while the KI and DPD concentrations were fixed at 26.1 mM and 1.1 mM, respectively. As shown in Figure 2a, the change in the green intensity (∆ green intensity) increased with the increasing AcOH concentration and then became constant at a 4.3 mM AcOH concentration, with the effect being more pronounced at higher PAA concentrations. This indicates a synergistic effect, where AcOH enhances the signal response for PAA detection. The ∆ green intensity under different AcOH and PAA concentrations (0.33 to 3.00 µg/mL), shown in Figure 2a, was used to create a PAA calibration curve. The sensitivity and linearity of the PAA calibration graph were plotted, as shown in Figure 2b. The results indicate that the sensitivity increased proportionally with the increase in the AcOH concentration, which ranged from 1.6 to 4.3 mM (Figure 2b; pink line). The sensitivity became constant at AcOH concentrations above 4.3 mM, following the graph relationship of the ∆ green intensity in Figure 2b. Additionally, the reaction at AcOH concentrations exceeding 17.4 mM occurred at a pH lower than 3.5, as shown in Figure S2, which is unsuitable for I^−^ oxidation, leading to a decrease in pink product formation. This may be due to the excessively low pH, which is unfavorable for I^−^ oxidation, as Awad et al. reported that the ideal pH for consistent I^−^ oxidation is between 3.5 and 5.4 [12]. An excellent linear correlation coefficient was also demonstrated when using 4.3, 5.4, and 6.5 mM AcOH. Thus, an AcOH concentration of 4.3 mM was selected for further investigation.

#### 2.2.2. Effect of I^−^ Concentration on Product Formation in PAA Determination

**Figure 3:**
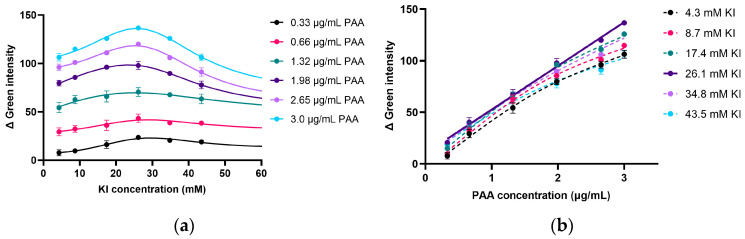
(**a**) Effect of KI concentration on relative green intensity at various PAA concentrations and (**b**) linear calibrations between PAA concentrations (0.33 to 3.0 µg/mL) and Δ green intensity obtained using different KI concentrations (*n* = 3).

I^−^ reacts with PAA to produce I_2_ (Equation (1)), which serves as an intermediate in DPD oxidation by PAA (Equation (2)). Therefore, the iodide concentration plays a crucial role in the reaction. The effect of the I^−^ concentration (4.3 to 43.5 mM) on DPD^•+^ formation was investigated. The DPD concentration was fixed at 1.1 mM while varying the PAA and I^−^ concentrations. Figure 3a presents the Δ green intensity of the pink-magenta product formed under varying I^−^ and PAA concentrations. The effect of the I^−^ concentration followed a similar trend, regardless of the different PAA concentrations. The increase in the pink-magenta product formation (DPD^•+^) was enhanced by the increasing I^−^ concentration, up to 26.1 mM. However, when the I^−^ concentration exceeded 26.1 mM, a decrease in pink-magenta product generation was observed. This may have been caused by the I_2_ formed (Equation (1)), which could have reacted with the excess I^−^ rather than with DPD (Equation (2)) to produce triiodide (Equation (3)).

I_2_ + I^−^ U+ ⬌ I_3_^−^ (3)

The highest relative green intensity was observed when using 26.1 mM KI, and the highest sensitivity of the calibration graph was found at 26.1 mM KI (Figure 3b).

#### 2.2.3. Effect of DPD Concentration on Product Formation

The effect of DPD concentration (0.4 to 8.7 mM) on the Δ green intensity of the product using PAA concentrations of 0.33 to 3.0 µg/mL was investigated. These experiments were conducted under the conditions of 26.1 mM KI and 4.3 mM AcOH. The results (Figure S3) indicate that, at a DPD concentration below 1.1 mM, the Δ green intensity was lower than that at higher DPD concentrations. This could be explained by the fact that, under conditions with elevated oxidant-to-DPD ratios, DPD undergoes oxidation and forms both DPD^•+^, the Würster dye (pink-magenta), and imine (colorless) products [18]. This dual oxidation explains the observed solution-fading phenomenon. However, at higher DPD concentrations (1.1 to 2.2 mM), the highest DPD^•+^ formation remained constant. Therefore, 1.1 mM of DPD is sufficient for the DPD^•+^ formation reaction and PAA determination.

#### 2.2.4. Investigation of the Period of DPD Oxidation by PAA

The period of DPD oxidation by PAA was investigated by monitoring the formation of the pink-magenta product under fixed concentrations of I^−^ (26.1 mM), AcOH (4.3 mM), and DPD (1.1 mM) across a range of PAA concentrations from 0.33 to 2.65 µg/mL. The incubation time varied from 0.5 to 5 min, following the addition of DPD. The sensitivity and linearity of the obtained calibration graph at each incubation time were examined. The sensitivity increased with longer incubation times, with no significant difference observed between 1 and 2 min. When the incubation time was further extended to 3 to 5 min, the sensitivity continued to improve; however, this was accompanied by a decrease in linearity. An incubation time of 1 min was selected due to the optimal sensitivity, linear range, and linearity. Moreover, a shorter incubation time could avoid the influence of several interference factors, such as H_2_O_2_, which could inhibit DPD oxidation, as reported by Pinkernell et al. [27].

### 2.3. Validation of the Custom-Built Mobile Application

**Figure 4:**
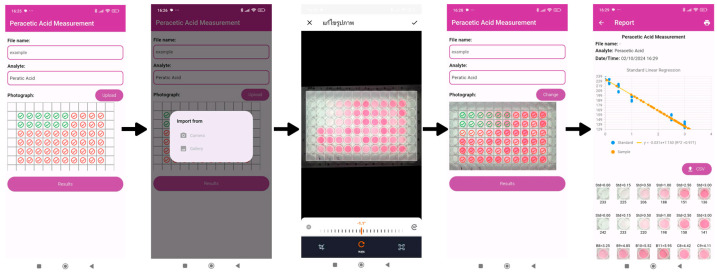
The *Modern Peracetic Acid Analysis* display window and the procedure for using the application.

A custom-built mobile application, named *Modern Peracetic Acid Analysis*, was developed by our laboratory for image processing to extract the color intensity and evaluate the PAA concentration in samples. The application is available online for Android (https://play.google.com/store/apps/details?id=com.analysislabcmu.paa (accessed on 18 May 2024). Figure 4 outlines the procedure for using the application. There are four steps: (i) filling out the sample details, (ii) uploading a photograph by taking one or importing one from a gallery, (iii) adjusting the scale to fit the layout, and (iv) pressing the results button for evaluation. The PAA concentration in each sample in a well is shown alongside images, and the results can be exported in CSV or PDF files. To validate the performance of *Modern Peracetic Acid Analysis*, a comparative analysis between our mobile application and common software/applications, including ImageJ (version 1.54d) and ColorMeter^®^ (version 2.2.0), was conducted. The obtained concentrations showed no statistically significant differences among the three analytical approaches at a 95% confidence interval. The percentage recoveries and relative standard deviations (RSDs), which are detailed in Table S1, all fell within an acceptable range.

### 2.4. Analytical Characteristics of the Proposed Approach for PAA Determination

#### 2.4.1. Linear Regression Analysis, Detection Limit, and Quantitation Limit

The proposed analytical procedure for PAA determination was characterized under optimal conditions using concentrations of 4.3 mM AcOH, 26.1 mM KI, and 1.1 mM DPD. The working linear range was found to be 0.15 to 3.0 µg/mL of PAA. The limit of detection and limit of quantitation were calculated as 0.11 µg/mL and 0.34 µg/mL, respectively, using the formulas 3.3σ and 10σ, where σ represents the standard deviation of the Y-intercept, divided by the slope of the linear calibration graph. Table 1 provides a comparison of the linear ranges, limits of detection (LODs), and limits of quantitation (LOQs) of the proposed approach, which are worse than those in previously reported procedures [12,21], but the method adequately covered PAA concentrations relevant to several real sample applications. The proposed approach exhibited a wider linear range. Similarly, in comparison to procedures using a different chromogenic reagent (ABTS) [28,29], the proposed approach also demonstrated a broader linear range.

#### 2.4.2. Accuracy and Precision

The accuracy of the proposed assay was evaluated through a spike study. Briefly, using a formulation containing a known PAA concentration, a series of five 10 mL mixtures was prepared by adding different volumes of a 10 µg/mL PAA standard into each, before making to the final volume (10 mL) with deionized water, to yield final concentrations of 0.5, 1.0, 1.5, 2.0, and 2.5 µg/mL PAA standard and 0.5 µg/mL PAA of formulation. The five mixtures had the same matrices. Triplicate PAA determinations were carried out for each mixture per period, with three periods per day for repeatability and over three separate days for intermediate precision assessments. A %recovery indicates accuracy, while %RSD indicates precision, as summarized in Table 2.

#### 2.4.3. Investigation of Interference of Active Compounds in Medical Formulations

The potential interference of active compounds commonly found in medical formulations, including I_2_, chlorhexidine, sodium hypochlorite, H_2_O_2_, AcOH, benzalkonium chloride, and dodecyl dimethyl ammonium chloride, was evaluated. The concentration ratios of interfering compounds to PAA observed in real samples were investigated based on reference ratios of 1% I_2_, 4% chlorhexidine, 6% hypochlorite, 10% H_2_O_2_, 20% AcOH, 80% benzalkonium chloride, and 80% didecyldimethylammonium chloride, relative to a PAA concentration of 5%. The percentage recovery was obtained to assess the influence of the interference. It was found that the percentage recovery was in the range of 92 ± 1% to 102 ± 3%, as shown in Table 3. These findings demonstrate that the proposed approach exhibits sufficient selectivity for real-world applications in medical fields.

### 2.5. Application in Medical Disinfectant Formulations as an Initial Model

The proposed approach was applied to determine the PAA concentrations in medical disinfectant formulations as an initial model of real application. Six samples were analyzed using the proposed approach and the acid–base neutralization titration method, which is a reference method based on the significantly different pKa values of PAA and acetic acid [7]. As shown in Table 4, no significant differences were observed between the results obtained using these two methods at a 95% confidence level. This indicates that the proposed approach is capable of quantitatively controlling the PAA concentration in pharmaceutical materials and disinfection products. Thus, the proposed approach could potentially promote a healthy life and well-being by reducing the risk of infections caused by ineffective sterilization, ensuring more reliable disinfection practices.

### 2.6. Green and Sustainable Assessment

**Figure 5:**
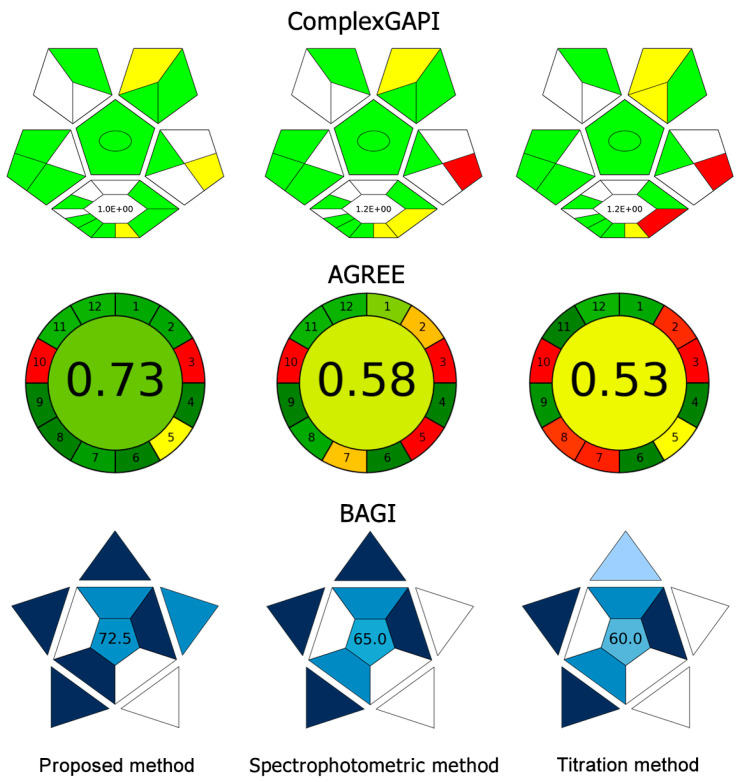
Degree of greenness of the proposed approach compared with the spectrophotometric and titration methods, assessed using the ComplexGAPI, AGREE, and BAGI.

The Analytical Greenness (AGREE) [30], Complementary Green Analytical Procedure Index (ComplexGAPI) [31], and Blue Applicability Grade Index (BAGI) [32] were employed to assess the environmental impact of the proposed approach in comparison to that of the titration [7] and spectrophotometric methods [21]. The degrees of greenness are illustrated in Figure 5. The input parameters used in the different models to assess greenness and sustainability are summarized in Table S2. According to the ComplexGAPI, the proposed approach is more environmentally friendly, largely due to its difference in the third and fourth pentagrams in terms of lower reagent consumption and waste generation, which, in turn, reduces the disposal and treatment costs. This aligns with the estimated greenness scores, ranging from 0 to 1, obtained using the AGREE tool based on the 12 principles of green analytical chemistry, which were 0.73, 0.58, and 0.53 for the proposed approach, spectrophotometric method, and titration method, respectively. The improved score of 0.20 and 0.15 compared to the titration and spectrophotometric methods should be due to a reduction in sample volume and waste generation, along with an increase in sample throughput (principal points 2, 7, and 8). The BAGI was used to complement the well-established green metrics by focusing primarily on the practical aspects of white analytical chemistry. The proposed approach has an advantage in terms of offering simple, portable instrumentation that is easy to operate.

**Figure 6:**
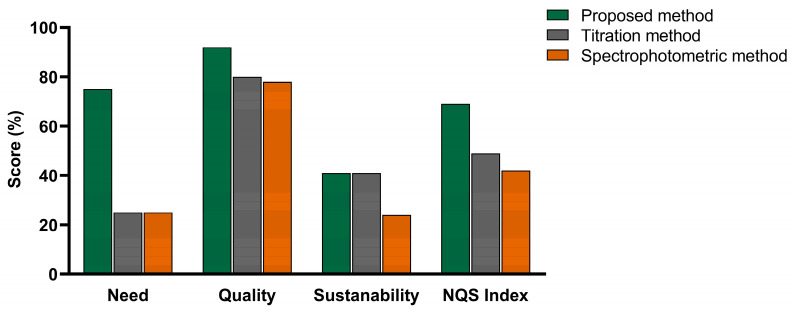
NQS index of the proposed approach compared with the titration and spectrophotometric methods.

Sustainability was further evaluated using the Need, Quality, and Sustainability (NQS) index [33], which considers factors such as cost, user requirements, and alignment with the Sustainable Development Goals (SDGs). As shown in Figure 6, the NQS index of the proposed approach is higher than that of the other methods, highlighting its superior sustainability. Beyond contributing to a healthy life and promoting well-being, the broader applicability of the proposed approach in fields such as agriculture and industry will further support additional SDGs, including improving nutrition and fostering sustainable agricultural practices.

## 3. Materials and Methods

### 3.1. Chemicals and Reagents

Peracetic acid was purchased from Thai Peroxide (Samutprakran, Thailand). Acetic acid, sodium hydroxide, and sulfuric acid were acquired from Merck Company (Darmstadt, Germany). Potassium iodide and ethylenediamine tetra-acetic acid were obtained from RCI Labscan Company (Bangkok, Thailand). N,N-Diethyl-p-phenylenediamine sulfate salt and benzalkonium chloride were purchased from Sigma-Aldrich Company (Darmstadt, Germany). Potassium hydrogen phthalate was obtained from KEMAUS Company (Cherrybrook, NSW, Australia). Iodine (Meiyume Manufacturing, Pathumthani, Thailand), sodium hypochlorite (Loba Chemie, Maharashtra, India), and didecyldimethylammonium chloride (Sigma-Aldrich Company, Buchs, Switzerland) were used.

### 3.2. Spectrophotometric Investigation of DPD-PAA Reaction

The absorption spectra of the DPD-PAA reaction mixture, containing 4.3 mM PAA, 26.1 mM KI, 4.3 mM AcOH, and 1.1 mM DPD combined with 2.0 mM ethylenediamine tetra-acetic acid (EDTA) in 0.5 M sulfuric acid at a volume ratio of 20:1:1:1, were measured using a microplate reader (EZ Read 2000, Biochrom, Cambridge, UK) at 380–800 nm.

### 3.3. Proposed Analytical Procedure

**Figure 7:**
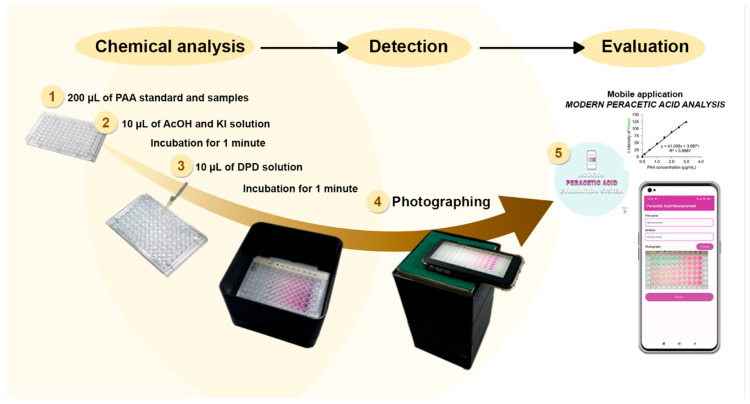
The proposed alternative green analytical procedure for PAA determination.

For the proposed procedure, PAA determination was carried out using a sterilized 96-well flat-bottom plate (Corning, New York, NY, USA) and a smartphone detector. In each well, 200 µL of the sample or standard was added, followed by 10 µL each of AcOH, KI, and DPD solutions. The optimal conditions, including reagent concentrations and oxidation times, were investigated, as described in Table S3. The plate was placed in a light-controlled box, and the resulting color generated under the optimum conditions was captured using a smartphone, with the flash turned off. The green intensity was extracted from the image and converted to a PAA concentration using a custom mobile application, named Modern Peracetic Acid Analysis. Figure 7 outlines the analytical procedure.

### 3.4. Development and Validation of the Custom-Built Mobile App

In this study, a custom-built mobile app, named *Modern Peracetic Acid Analysis*, was developed by our laboratory to extract the color intensity and evaluate the PAA concentration in samples. The accuracy of the application’s color intensity extraction and concentration evaluation was investigated by comparing these values with those determined via commercial image analysis software, including ImageJ (version 1.54d) and ColorMeter^®^ (version 2.2.0), in conjunction with Microsoft Excel. Blind samples (four concentrations) were prepared and measured using the proposed analytical procedure. The PAA concentrations were evaluated using image processing techniques across the three approaches, and the obtained concentration, the percentage of recovery, and the RSDs were compared.

### 3.5. Reference Method and Statistical Analysis

Classic acid–base titration based on the pKa was used to measure the PAA concentration [7] in medical disinfectant products. A 50 mL sample was titrated with 0.25 M sodium hydroxide solution (NaOH) using a pH meter (PH700 Benchtop, Apera Instruments, Shanghai, China). NaOH was standardized with a 1.0 M potassium hydrogen phthalate (KHP) solution before use.

All experiments were performed in triplicate. Paired *t*-test analyses were used to compare sample results obtained from the proposed and reference procedures. The statistical calculations were based on a 95% confidence level.

## 4. Conclusions

A simple, rapid, and green alternative approach for PAA determination, based on a colorimetric reaction involving DPD oxidation by PAA via an intermediate reaction with KI, was successfully developed. The effectiveness of this approach was demonstrated by determining the PAA concentration in pharmaceutical disinfectant samples at ready-to-use concentrations without any influence of other factors that may be present in the samples. Thus, the proposed approach could help ensure proper sterilization and adherence to safety standards. The developed portable platform, integrated with a smartphone, enables low-cost, on-site analyses. Photographs and data files acquired through mobile applications can be seamlessly shared via communication networks, facilitating collaboration among teams. Additionally, the information can be stored for future retrieval and traceability as needed. This initial model for real-world application promotes sustainable pharmaceutical analyses. Moreover, this approach can be expanded to the food and beverage industries and cattle farming, supporting sustainable practices in both industry and agriculture.

## Figures and Tables

**Table 1:** Analytical characteristics of the proposed approach in comparison to those of previous spectrophotometric methods.

Detection Technique	Chromogenic Agent	Range (µg/mL)	R^2^	LOD (µg/mL)	LOQ (µg/mL)	References
Image processing	DPD	0.15–3.0	0.9974	0.11	0.34	Proposed approach
Spectrophotometric method	DPD	0.10–1.65	0.9967	-	-	[12]
		0.125–2.5	0.9944	0.0015	0.0025	[21]
	ABTS	0.04–0.76	0.9999	0.0008	-	[28]
		0.16–1.2	0.999	0.0023	-	[29]

**Table 2:** Accuracy and precision of PAA determination.

PAA Concentration Spiked (µg/mL)	Repeatability						Intermediate Precision	
	Day1		Day2		Day3			
	%Recovery	%RSD	%Recovery	%RSD	%Recovery	%RSD	%Recovery	%RSD
0.5	98 ± 5	5	105 ± 4	3	101 ± 4	4	102 ± 4	3
1.0	99 ± 2	2	94 ± 3	3	102 ± 2	2	98 ± 4	4
1.5	100 ± 5	5	102 ± 5	5	101 ± 2	2	101 ± 1	1
2.0	104 ± 5	5	102 ± 5	5	100 ± 1	1	102 ± 3	2
2.5	104 ± 1	1	99 ± 4	4	97 ± 1	1	100 ± 4	4

**Table 3:** Percentage recovery of spiked PAA concentration in commercial disinfectants containing different active compounds and other components.

Major Interfering Substances in Disinfectant	%Recovery
Iodine	95 ± 1
Chlorhexidine	92 ± 1
Hypochlorite	94 ± 3
Hydrogen peroxide	97 ± 1
Acetic acid	94 ± 1
Benzalkonium chloride	102 ± 3
DDAC ^1^	93 ± 2

^1^ DDAC is didecyldimethylammonium chloride.

**Table 4:** Comparison of the proposed approach and titration to determine PAA in samples.

No.	Titration		Proposed Approach	
	PAA Concentration (%)	%RSD	PAA Concentration (%)	%RSD
S1	0.14 ± 0.000	0.0	0.16 ± 0.002	1.5
S2	0.15 ± 0.001	0.7	0.16 ± 0.004	2.4
S3	0.18 ± 0.003	1.7	0.18 ± 0.006	3.2
S4	5.84 ± 0.003	0.1	5.81 ± 0.120	2.1
S5	4.56 ± 0.000	0.0	4.44 ± 0.093	2.1
S6	0.15 ± 0.000	0.0	0.15 ± 0.002	1.3

## Data Availability

The data presented in this study are available on reasonable request from the corresponding author.

## Supplementary Materials

The following supporting information can be downloaded at https://www.mdpi.com/article/10.3390/molecules30132798/s1: Figure S1: The relationship between the PAA concentrations (0.33 to 2.65 µg/mL) and Δ intensity of red, green, and blue; Figure S2: pH of the mixture under different AcOH conditions. Experimental conditions: [PAA] = 3.31 µg/mL, [DPD] = 1.1 mM, [AcOH] = 0, 2.2, 4.3, 8.7, and 17.4 mM, [I^−^]_initial_ = 26.1 mM, and reaction time = 60 s; Figure S3: Effect of DPD concentration on the relative green intensity of DPD^•+^ at different PAA concentrations; Table S1: Percentages of recovery and relative standard deviation from the blind sample determination; Table S2: Parameters for assessing greenness and sustainability; Table S3: Parameters for the optimum conditions for PAA determination.

## Abbreviations

The following abbreviations are used in this manuscript:

AcOH	Acetic acid
ABTS	bis(3-ethylbenzothiazoline-6-sulfonate)
DPD	N,N-diethyl-p-phenylenediamine
KHP	Potassium hydrogen phthalate
PAA	Peracetic acid
LODs	Limits of detection
LOQs	Limits of quantitation
AGREE	Analytical Greenness
ComplexGAPI	Complementary Green Analytical Procedure Index
BAGI	Blue Applicability Grade Index
NQS index	Need, Quality, and Sustainability Index
IPC	Infection Prevention and Control
RSDs	Relative Standard Deviations
SDGs	Sustainable Development Goals
