# Supplementary material for: Portable and Rapid Smartphone-Based Colorimetric Assay of Peracetic Acid for Point-of-Use Medical/Pharmaceutical Disinfectant Preparation
Source: Molecules. 2025 Jun 28;30(13):2798. doi: 10.3390/molecules30132798 (PMC12250838; doi:10.3390/molecules30132798)
Supplement: Supplementary file 1 [file molecules-30-02798-s001.zip › molecules-3687783-supplementary.pdf]

molecules

MDPI

Article

Portable and rapid smartphone-based colorimetric assay of peracetic acid for point-of-use medical/pharmaceutical disinfectant preparation

Suphakorn Katib [1,3], Sutasinee Apichai [2,3], Jutamas Jiaranaikulwanitch [1,3], Busaban Sirithunyalug [1,3], Fumihiko Ogata [4], Naohito Kawasaki [4,5], Kate Grudpan [3,6] and Chalermpong Saenjum [1,3,*]

[1] Department of Pharmaceutical Sciences, Faculty of Pharmacy, Chiang Mai University, Chiang Mai 50200, Thailand; suphakorn2801@gmail.com (S.K.); jutamas.jia@cmu.ac.th (J.J.); busaban.s@cmu.ac.th (B.S.); chalermpong.s@cmu.ac.th (C.S.)
[2] Office of Research Administration, Chiang Mai University, Chiang Mai, 50200, Thailand; sutasinee.apichai@cmu.ac.th (S.A.)
[3] Research Center for Innovation in Analytical Science and Technology for Biodiversity Based Economic and Society (I-ANALY-S-T_B.BES-CMU), Chiang Mai University, Chiang Mai 50200, Thailand
[4] Faculty of Pharmacy, Kindai University, 3-4-1 Kowakae, Higashi-Osaka, Osaka, 577-8502, Japan; ogata@phar.kindai.ac.jp (F.O.); kawasaki@phar.kindai.ac.jp (N.K.)
[5] Antiaging Center, Kindai University, 3-4-1 Kowakae, Higashi-Osaka, Osaka, 577-8502, Japan
[6] Department of Chemistry, Faculty of Science, Chiang Mai University, Chiang Mai, Thailand; kgrudpan@gmail.com (K.G.)
* Correspondence: chalermpong.s@cmu.ac.th; Tel.: +66-89-950-4227

Supplementary Material

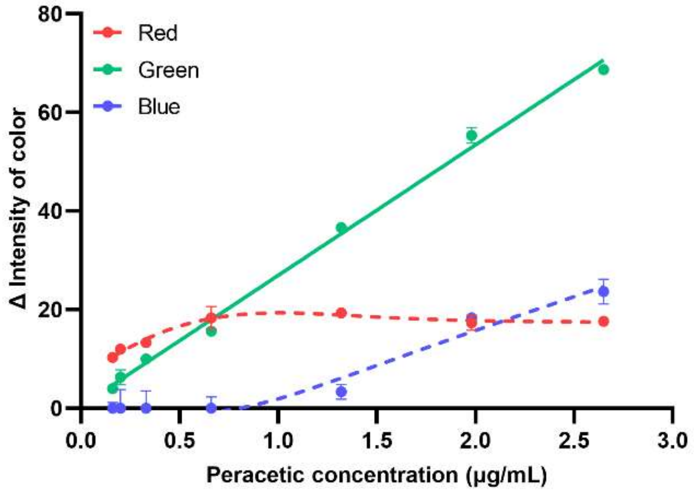

Figure S1. The relationship between the PAA concentrations (0.33 to 2.65 μg/mL) and Δ intensity of red, green, and blue.

 https://doi.org/10.3390/xxxxx

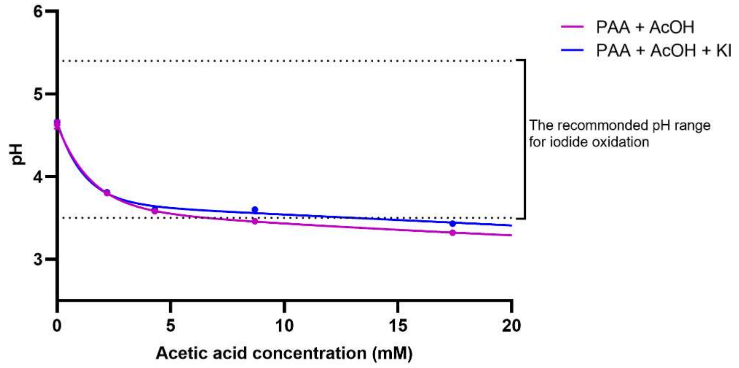

Figure S2. pH of the mixture under different AcOH conditions. Experimental conditions: [PAA] = 3.31 µg/mL, [DPD] = 1.1 mM, [AcOH] = 0, 2.2, 4.3, 8.7, and 17.4 mM, $[I^-]$initial = 26.1 mM, and reaction time = 60 s.

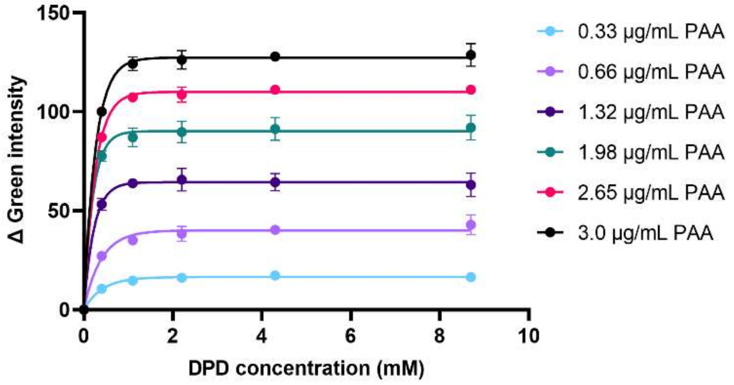

Figure S3. Effect of DPD concentration on the relative green intensity of $DPD^{•+}$ at different PAA concentrations

Table S1. Percentages of recovery and relative standard deviation from the blind sample determination.

Blind sample	Concentration of blind sample (µg/mL)	ColorMeter® (n=3)		ImageJ (n=3)		Modern Peracetic Acid Analysis (n=3)	
		%Recovery	%RSD	%Recovery	%RSD	%Recovery	%RSD
1	1.5	96	2	93	1	94	1
2	2.0	100	3	102	3	102	3
3	2.5	102	2	102	2	100	3
4	3.0	101	2	100	3	101	1

Table S2. Parameters for assessing greenness and sustainability

Parameters	Proposed method	Spectrophotometric method	Titration method
Sample (mL)	0.2	7	40
Reagent (mL/sample)	0.03	0.3	5
Waste (mL/sample)	0.23	7.3	45
Energy for instrumentation (KWh)	≤1	≤1	≤1
Sample throughput (samples/hr in duplicate)	240	30	6
Instrumentation	Simple operation, portable instrument	Simple instrumentation, available in most laboratories	

Table S3. Parameters for the optimum conditions for PAA determination.

Condition	Parameter	Value
1	KI (mM)	8.7, 17.4, 26.1, 34.8, 43.5
	DPD concentration (mM)	1.1
	AcOH concentration (mM)	4.3
	Incubation time (min)	1
2	KI (mM)	26.1
	DPD concentration (mM)	1.1
	AcOH concentration (mM)	2.2, 4.3, 8.7, 17.4, 26.1, 34.8, 43.5
	Incubation time (min)	1
3	KI (mM)	26.1
	DPD concentration (mM)	0.4, 1.1, 2.2, 4.3, 8.7
	AcOH concentration (mM)	4.3
	Incubation time (min)	1
4	KI (mM)	26.1
	DPD concentration (mM)	1.1
	AcOH concentration (mM)	4.3
	Incubation time (min)	0.5, 1, 2, 3, 4, 5
